# Impact of the Chelsea critical care physical assessment (CPAx) tool on clinical outcomes of surgical and trauma patients in an intensive care unit: An experimental study

**DOI:** 10.4102/sajp.v74i1.450

**Published:** 2018-08-23

**Authors:** Megan Whelan, Heleen van Aswegen, Evelyn Corner

**Affiliations:** 1 Department of Physiotherapy, School of Therapeutic Sciences, Faculty of Health Sciences, University of the Witwatersrand, South Africa; 2 Department of Clinical Sciences, Brunel University London, United Kingdom; 3 Centre for Human Performance, Exercise and Rehabilitation, Brunel University London, United Kingdom; 4 Chelsea and Westminster NHS Foundation Trust, London, United Kingdom

## Abstract

**Background:**

Critically ill patients following traumatic injury or major surgery are at risk of loss of skeletal muscle mass, which leads to decreased physical function. Early rehabilitation in an intensive care unit (ICU) is thought to preserve or restore physical functioning. The Chelsea critical care physical assessment (CPAx) is a measurement tool used to assess physical function in the ICU.

**Objectives:**

To determine whether the use of the CPAx tool as part of physiotherapy patient assessment, in two adult trauma and surgical ICU settings where early patient mobilisation forms part of standard physiotherapy practice, had an impact on ICU and hospital length of stay (LOS) through delivery of problem-oriented treatment plans.

**Method:**

A single-centred pre–post quasi-experimental study was conducted. The population was a consecutive sample of surgical and trauma ICU patients. Participants’ functional ability was assessed with the CPAx tool on alternative days during their ICU stay, and rehabilitation goals were modified according to their CPAx score. Intensive care unit and hospital LOS data were collected and compared to data of a matched historical control group. Descriptive and inferential statistics were used.

**Results:**

A total of 26 ICU patients were included in the intervention group (*n* = 26). They received CPAx-guided therapy, and outcomes were matched with ICU patients in the historical control group (*n* = 26). The median sequential organ failure assessment (SOFA) score was significantly higher in the control group (*p* = 0.005) (3.5 [IQR 2–6.3]) versus (2 [IQR 1.8–2.5]) for the intervention group. The median admission CPAx score for the intervention group was 33.5 (IQR 16.1–44), and the median ICU discharge score was 38 (IQR 28.5–43.8). No significant differences were found in ICU days (control 2.7 [IQR 1.1–5.2]; intervention 3.7 [IQR 2.3–5.4]; *p* = 0.27) or hospital LOS (control 13.5 [IQR 9.3–18.3]; intervention 11.4 [IQR 8.4–20.3], *p* = 0.42). Chelsea critical care physical assessment scores on ICU admission had a moderate negative correlation with hospital LOS (*r* = −0.58, *p* = 0.00, *n* = 23). Chelsea critical care physical assessment scores at ICU discharge had strong positive correlation with discharge SOFA scores (*r* = 0.7; *p* = 0.025; *n* = 10).

**Conclusion:**

Problem-oriented patient rehabilitation informed by the CPAx tool resulted in improvement of physical function but did not reduce ICU or hospital LOS.

**Clinical implications:**

A higher level of physical function at ICU admission, measured with CPAx, was associated with shorter hospital LOS.

## Introduction

The stress response to major surgery and trauma, including hormonal and metabolic changes, as well as an increase in cytokine production following cell injury (Desborough 2000), can render a patient critically ill. The incidence of trauma has escalated worldwide over the past decade (World Health Organisation [WHO], 2014). Interpersonal violence was the fourth leading cause of death in young adults in 2014, and road traffic collisions is projected to be the seventh leading cause of death worldwide by 2030 (WHO 2014, 2016); all of these patients are at high risk of critical illness. Additionally, elective major surgery for conditions such as cancerous disease, gynaecological complications or wound infections can result in a similar stress response and critical illness. In low- and middle-income countries, such as South Africa, the acute physiology and chronic health evaluation (APACHE) II and the sequential organ failure assessment (SOFA) scores are recommended for use to determine a patient’s risk of hospital mortality, rate of organ failure and prognosis (Haniffa et al. 2018; Shabir & Maqbool 2017). The likelihood of major surgery leading to critical illness is increased in older adults with comorbidities or malnutrition (Farhan et al. 2016). Once critical illness sets in, there is a high risk of rapid muscle wasting, which can render a patient fully dependent (Corner et al. 2014). From a surgical perspective (be that elective or emergency surgery following trauma), the surgery performed, type of drugs administered to the patient during surgery and the duration of surgery impact on skeletal muscle mass (Farhan et al. 2016). Subsequent patient management in the ICU, specifically patient sedation practices, enforced bedrest and the onset of sepsis, can contribute to further loss of skeletal muscle and lead to the development of ICU-acquired weakness (ICU-AW) (Farhan et al. 2016). ICU-acquired weakness is a common debilitating condition associated with prolonged critical illness (Saxena & Hodgson 2012). The incidence of ICU-AW in the surgical ICU population is as high as 74% (Farhan et al. 2016; Hermans & Van den Berghe 2015; Saxena & Hodgson 2012). Common complications linked with ICU-AW include difficulty with weaning from mechanical ventilation, increased hospital length of stay (LOS), long-term functional impairments and disability, as well as reduced health-related quality of life (Corner 2012; Farhan et al. 2016; Hermans & Van den Berghe 2015). Some evidence suggests that early progressive mobilisation and rehabilitation of patients in the ICU is an important strategy to combat the negative effects associated with critical illness and immobility, in order to improve patients’ functional ability and reduce ICU and hospital LOS (Kayambu, Boots & Paratz 2013; Stiller 2013).

Marques, Bruton and Barney (2006) stated that outcome measures are vital to determine patients’ responses to treatment received, in order to evaluate the usefulness of the treatment and to allow for comparison between interventions. However, clinical utility of outcome measures is still low. Maher and Williams (2005) found that only 17% of physiotherapists caring for lung transplant patients used outcome measures as part of their routine care – this was because of time constraints and lack of equipment. Similarly, functional outcome measures are not consistently used by physiotherapists in the critical care settings (Corner 2012; Corner, Handy & Brett 2016). This may be because they are considered time consuming and in the past lacked specificity in the ICU population (Corner 2012). Subsequently, a number of ICU-specific measures have been developed (Corner et al. 2013; Denehy et al. 2013; Sommers et al. 2016).

A patient’s ability to perform functional activities in ICU is dependent on their alertness, balance, cognition and strength (Corner et al. 2014). In surgical and trauma ICU populations, inflammatory responses because of the injuries sustained, surgical procedures performed and severity of illness impact negatively on skeletal muscle mass and may affect a patient’s ability to perform functional activities (Farhan et al. 2016; Hermans & Van den Berghe 2015; Saxena & Hodgson 2012). The Chelsea critical care physical assessment (CPAx) tool is an outcome measure designed to assess 10 domains of physical ability: respiratory function, cough, bed mobility, supine to sitting on the edge of the bed, dynamic sitting, sit to stand, standing balance, transferring from bed to chair, stepping and grip strength (Corner et al. 2013).

The tool is simple to use, responsive to change and has minimal floor and ceiling effects (Corner et al. 2014, 2015). It was designed to be used daily to monitor patients’ function and identify problem areas (Corner et al. 2013, 2016). Once problem areas have been identified, a specific rehabilitation programme can be tailored to the needs of the patient (Corner et al. 2013). Therefore, theoretically, the use of the CPAx may facilitate problem-oriented treatment plans and enhance clinical care.

## Aims and objectives

The primary aim of this study was to determine whether the use of the CPAx tool as part of physiotherapy patient assessment, in two adult trauma and surgical ICU settings where early patient mobilisation forms part of standard physiotherapy practice, had an impact on ICU and hospital LOS through delivery of problem-oriented treatment plans.

*Null hypothesis*: There is no difference in LOS in the adult trauma and surgical ICU populations following implementation of the CPAx tool as a method of ensuring problem-oriented treatment plans into clinical practice.

Secondary objectives were (1) to measure change in physical function in ICU patients using the CPAx tool and (2) to establish the relationship between CPAx scores and severity of illness and morbidity scores during ICU stay as a means of discriminant validation.

## Methodology

The principles for conducting research on human subjects, outlined in the Declaration of Helsinki, were adhered to throughout the duration of the trial.

A pre–post quasi-experimental design with a historical matched control group was used. The study was conducted at Chris Hani Baragwanath Academic Hospital (CHBAH), a provincial public sector hospital situated in Soweto, South Africa. This is the third largest hospital in the world with a catchment area of 161 km (https://www.chrishanibaragwanathhospital.co.za/). The emergency department personnel manage on average 650 000 patients per year, including approximately 160 victims of gunshot wounds per month (https://www.chrishanibaragwanathhospital.co.za/). The ICU is divided into two units: a trauma ICU and a general surgical ICU. Within the CHBAH ICUs, there is a culture of minimising sedation and early mobilisation. The dietitians review patients daily to maintain nutritional status. Daily multidisciplinary ward rounds ensure that there is good communication between the physiotherapists, doctors, nurses and dietitians.

### Intervention group

All adult patients admitted into the trauma ICU and surgical patients admitted into the general ICU (October 2015–February 2016) were considered for inclusion in the prospective phase of the study through the use of consecutive sampling. Those not considered for inclusion were bedbound patients prior to admission, traumatic brain injury or surgery received for other neurological conditions, those placed on bed rest in ICU as a result of complex orthopaedic or spinal injuries sustained and those admitted to the general or trauma ICU one month prior to the start of the pilot study, during which time clinicians familiarised themselves with the CPAx tool.

### Control group

A retrospective review of patient admissions into the trauma and general ICUs (01 January 2014 – 30 June 2015) was completed to identify patients who could be matched with those included in the intervention group. Participants were matched according to age categories, gender, matching of diagnoses and APACHE II scores. Age categories were defined in years as 20–29, 30–39, 40–49, 50–59 and 60–69. Matching of diagnoses was performed according to the nature of the injuries sustained (for patients in the trauma ICU) as well as surgical procedures that were performed (for the patients in the general ICU). Historical control participants were considered a potential match if their APACHE II scores were within three points of the APACHE II scores for the participants recruited into the intervention group. Participants had to match with the intervention group against all four criteria to be included.

The sample size was calculated based on LOS data from the general and trauma ICUs from December 2014 – February 2015. The mean ICU LOS during this period was 5.8 days (standard deviation [SD] 5.9; *n* = 24). The mortality rate in this sample of patients was 37.5% (*n* = 9). A minimum sample size of 11 participants per group (95% C.I. ± 3.7) was determined to detect a 12-h difference in ICU LOS (SD of 5.9, α 0.05, non-compliance 10%, drop out [including mortality] 38% at 1-β 90%). Recruitment of the intervention group was completed between October 2015 and February 2016.

## Measurement instruments

Three data collection instruments were used, namely the APACHE II and SOFA scores and the CPAx. A short description of each instrument follows below.

### Acute physiology and chronic health evaluation II score

The APACHE scoring system is a well-recognised and widely used severity of disease classification system used in ICU to predict the risk of hospital mortality (Haniffa et al. 2018). In a recent review, it was reported to have good to excellent ability to discriminate between survivors and non-survivors at ICU discharge in low- to middle-income countries (Haniffa et al. 2018). A higher score (maximum score is 71) reflects a higher severity of illness and a higher risk of mortality. The ICUs at CHBAH use the APACHE II system to record patient severity of illness information.

### Chelsea critical care physical assessment tool

The CPAx was used to measure physical function in ICU. Each of the 10 domains of the CPAx tool is graded from 0 (complete dependence) to 5 (complete independence) (Corner et al. 2014). A total score out of 50 is then obtained and is depicted in pictorial form on a ‘radar’ chart. The CPAx tool has been shown to be a valid tool in the assessment of physical morbidity and is effective in detecting changes in physical function (Corner 2012; Corner et al. 2015). It has a limited floor and ceiling effect, and reliability testing has shown that CPAx has strong internal consistency and inter-rater reliability (Corner et al. 2013, 2015, 2016). A higher score represents greater functional ability.

### Sequential organ failure assessment score

This tool is widely used in ICUs globally to assess a patient’s severity of organ failure during their ICU stay. It can be completed daily to track change and is also an indicator of prognosis (Shabir & Maqbool 2017). The SOFA score has good reliability and is accurate in predicting patients’ outcome (Arts et al. 2005; Oliveira-Neto et al. 2012; Shabir & Maqbool 2017). An increase in SOFA score in the first 96 h of admission relates to a mortality rate of at least 50% (Ferreira et al. 2001).

## Procedure

Four physiotherapists work clinically in the two participating ICUs: two permanent and two rotational. Only the two permanent physiotherapists participated in the study and acted as research assistants. Prior to data collection, they completed the online CPAx training module (https://cpax.helmlms.com/) developed by E.J. Corner, before they were allowed to administer the tool. This was followed by a pilot study to determine the inter-rater reliability of CPAx scores obtained between the two research assistants during patient assessments. Excellent inter-rater reliability (ICC = 0.999; 95% CI = 0.994–1) was demonstrated, and no changes to the study procedure were required.

### Historical control group

The first author (M.W.) was responsible for searching the ICU databases at CHBAH to find appropriately matched patients for the control group. The control group had received standard physiotherapy care during their ICU stay in 2014–2015. This included a standard initial ICU assessment and interventions such as a variety of chest physiotherapy techniques (as indicated), graduated bed exercise programmes and early mobilisation strategies for those stable enough to participate. At this stage, no outcome measures to assess physical function were being used by the physiotherapists. Information obtained for matched patients was captured by M.W. on the study recording sheets and transferred into electronic format for data analysis.

### Intervention group

The two permanent physiotherapists (research assistants) performed daily screening of consecutive new patient admissions to identify potential participants according to the study criteria. Once participants had been identified, written informed consent was gained from willing participants. Participants were not randomly allocated to the research assistants. Participant assessment involved a standard initial ICU assessment, followed by a CPAx assessment. A Smedley^®^ digital hand-grip dynamometer was used to assess participants’ grip strength (as required for the CPAx tool) (Corner et al. 2013). Participants received daily (or twice daily, if indicated) physiotherapy care (similar to that described above for the control group) with rehabilitation targeted at problems identified using the CPAx. Chelsea critical care physical assessment and SOFA (using a free online SOFA calculator) scores for participants in the intervention group were calculated and recorded by the two trained physiotherapists on alternate weekdays during their ICU stay. Once participants were discharged to the ward, they continued to receive physiotherapy management; however, CPAx assessments were discontinued. A different team of physiotherapists took over the care of the patients once they were discharged from ICU. The specific treatment given to the study participants following ICU discharge was at the discretion of the treating physiotherapist.

## Statistical analysis

Per protocol analysis was performed using the IBM^®^ Statistical Package for Social Sciences (SPSS) version 24 for Windows software. Descriptive statistics were used to present the data. Normality was tested using the Shapiro–Wilk test. Categorical data were summarised as frequencies and percentages. Continuous data are presented as mean, median, standard deviations (SD) and interquartile range (IQR), depending on normality. Between groups comparisons were made using the Mann–Whitney U test for non-parametric data, and the independent *t*-test for parametric data. Spearman’s correlation coefficient was used to determine if a relationship existed between CPAx scores, LOS, APACHE II scores and SOFA scores. A *p* ≤ 0.05 was deemed statistically significant.

### Ethical considerations

Permission to conduct this study was obtained from the University of the Witwatersrand Human Research Ethics (Medical) Committee (certificate number: M150726), Johannesburg, South Africa, and the local institutional review board.

## Results

**FIGURE 1:**
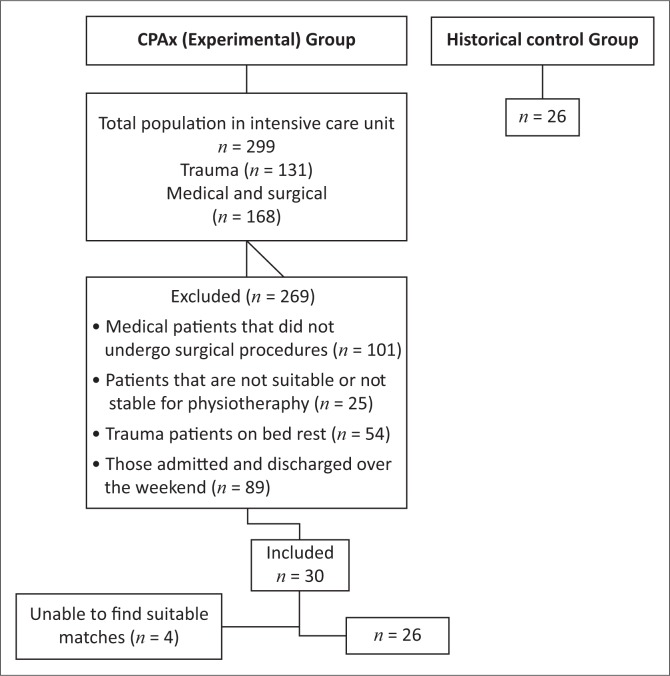
Summary of participant recruitment for the study.

Participant recruitment and attrition is summarised in Figure 1. During the study period, 30 participants were recruited into the intervention group. Four participants were excluded because of a lack of suitable matched controls. Twenty-six participants were recruited into the historical control group. One participant in the intervention group died during his stay in the ICU. Two participants died in the wards, and one was transferred to another hospital.

Participant characteristics are summarised in Table 1. The groups were comparable for baseline characteristics such as age, gender, diagnoses and APACHE II score at ICU admission. The median SOFA score at ICU admission was significantly lower for the intervention group than for the control group (*p* = 0.005); however, there was no significant difference in SOFA scores at ICU discharge. There was no significant difference in median ICU LOS in days between groups (intervention 3.7 [2.3–5.4]; control 2.7 [IQR 1.1–5.2]; *p* = 0.27). There was no significant difference in median hospital LOS between groups (intervention 11.4 [IQR 8.4–20.3]; control 13.5 [9.3–18.3]; *p* = 0.42). The majority of participants in the trauma group suffered penetrating injury to the thorax and abdomen. Those in the surgical group mostly underwent surgery for gynaecological reasons or for removal of cancerous growths.

**TABLE 1:** Characteristics of study participants.

Characteristics	Intervention group (*n* = 26)	Control group (*n* = 26)	*p*
**Age (years) (median, IQR)**	33 (27–44.5)	35.5 (27–45.8)	0.900
**Gender *n* (%)**			1.000
Male	17 (65.4)	17 (65.4)	
Female	9 (34.6)	9 (34.6)	
**APACHE II scores (mean, SD)**	11.6 (5.2)	12 (5.0)	0.810
**Diagnosis *n* (%)**			1.000
Surgical	14 (53.8)	14 (53.8)	
Trauma	12 (46.2)	12 (46.2)	
**Type of surgery *n* (%)**			-
Adrenal mass resection	1 (7.1)	0 (0.0)	
Caesarean section	6 (42.8)	6 (42.8)	
Debridement for necrotising fasciitis	1 (7.1)	1 (7.1)	
Head and neck exploration for cancer	2 (14.3)	2 (14.3)	
Laparotomy for complicated appendicectomy	1 (7.1)	0 (0.0)	
Laparotomy for bowel disease	0 (0.0)	2 (14.3)	
Laparotomy for pancreatic cancer	0 (0.0)	1 (7.1)	
Nephrectomy	1 (7.1)	1 (7.1)	
Total colectomy	1 (7.1)	0 (0.0)	
Whipples procedure	1 (7.1)	1 (7.1)	
**Type of trauma *n* (%)**			-
GSW abdomen	5 (41.6)	5 (41.6)	
Injury to extremities	2 (16.6)	1 (8.3)	
Multiple rib fractures	1 (8.3)	1 (8.3)	
Stabbed heart	2 (16.6)	3 (25)	
Stabbed neck	1 (8.3)	1 (8.3)	
Stabbed thorax	3 (25)	2 (16.6)	
**SOFA scores (median, IQR)**			
Admission	2 (1.8–2.5)	3.5 (2–6.3)	0.005*
ICU discharge	2 (1.8–2)	2.5 (2–3)	0.060
**ICU LOS (days) (median, IQR)**	3.7 (2.3–5.4)	2.7 (1.1–5.2)	0.270
**Hospital LOS (days) (median, IQR)**	11.4 (8.4–20.3)	13.5 (9.3–18.3)	0.420

SD, standard deviation; APACHE, acute physiology and chronic health evaluation; SOFA, sequential organ failure assessment; GSW, gunshot wound; IQR, interquartile range.

* , *p* ≤ 0.05.

**FIGURE 2:**
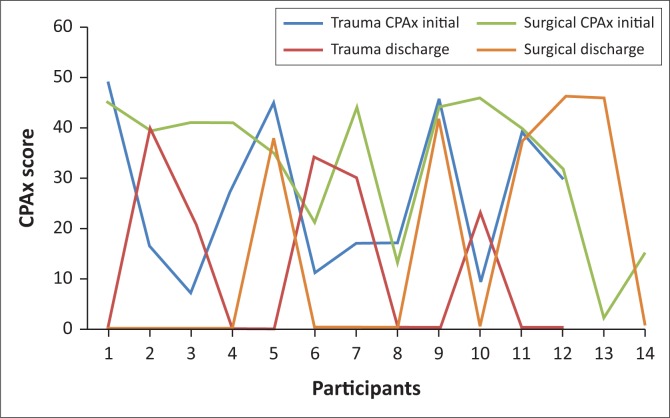
Differences in Chelsea critical care physical assessment (CPAx) scores at intensive care unit admission and discharge between participants following surgery or trauma.

The median CPAx score at ICU admission for the intervention group (*n* = 26) was 33.5 (IQR 16.1–44; range 2–40). The median CPAx score at ICU discharge (*n* = 10) was 38 (IQR 28.5–43.8; range 23–46). The difference in median CPAx scores between ICU admission and ICU discharge was 4.5 for the intervention group. Table 2 summarises differences in CPAx scores between the surgical and trauma populations. The median CPAx score at ICU admission was higher for the surgical group than the trauma group but this did not reach significance (*p* = 0.35). Because of the high turnover of patients in the trauma and general ICUs of CHBAH, and the fact that CPAx assessments were conducted on alternate days, only 10 participants had CPAx assessments at ICU discharge. Five participants (50%) were surgical cases and five (50%) were trauma cases. The difference in median CPAx scores at ICU discharge between the surgical and trauma groups was 13 (*p* = 0.028) in favour of the surgical group (Figure 2). From ICU admission to discharge, the median CPAx scores changed by 3.2 points for the surgical group and by 7.5 points for the trauma group. Changes in functional ability for participants in the trauma group were therefore greater than for the surgical group, as measured with CPAx.

**TABLE 2:** Differences in Chelsea critical care physical assessment scores between surgical and trauma participants.

Diagnosis category	Number of participants	Median (IQR)	Minimum	Maximum	*p*
**CPAx scores on ICU admission**					0.350
Surgical cases	14	39.8 (19.5–44)	2	46	
Trauma cases	12	22.5 (12.4–43.5)	7	49	
**CPAx scores at ICU discharge**					0.028*
Surgical cases	5	43 (38–46)	37	46	
Trauma cases	5	30 (23.5–36.8)	23	40	

IQR, interquartile range; ICU, intensive care unit; CPAx, Chelsea critical care physical assessment.

* , *p* < 0.05.

CPAx scores on ICU admission had a moderate negative correlation with hospital LOS (*r* = −0.58, *p* = 0.001, *n* = 23). There was no correlation between CPAx at admission and ICU LOS (*r* = −0.19, *p* = 0.38, *n* = 23) or between CPAx at ICU discharge and ICU LOS (*r* = −0.58, *p* = 0.13, *n* = 8) or hospital LOS (*r* = −0.11, *p* = 0.78, *n* = 8). There was no correlation between APACHE II and CPAx scores on admission (*r* = −0.07, *p* = 0.72, *n* = 26) or with CPAx scores at ICU discharge (*r* = 0.02, *p* = 0.96, *n* = 10). Admission SOFA scores had a weak negative correlation with admission CPAx scores (*r* = −0.37, *p* = 0.06, *n* = 26). Chelsea critical care physical assessment scores at ICU discharge had a strong positive correlation with SOFA scores at ICU discharge (*r* = 0.7, *p* = 0.025, *n* = 10).

## Discussion

The aim of this study was to establish whether the use of the CPAx tool as part of physiotherapy patient assessment, in two adult trauma and surgical ICU settings where early patient mobilisation forms part of standard physiotherapy practice, had an impact on ICU and hospital LOS through delivery of problem-oriented treatment plans. The results support the null hypothesis as no difference in ICU or hospital LOS was found following implementation of the CPAx tool as a method of ensuring problem-oriented treatment plans into clinical practice.

Physical function improved between admission and discharge from ICU for all participants in the intervention group. Participants in the surgical group demonstrated significantly better physical function at ICU discharge than those in the trauma group. Even though physical function improved, it did not impact on ICU or hospital LOS when compared to historical control group results. Changes in physical function are expected to occur during the course of a patient’s journey after ICU admission. Improvement in functional ability may be quicker for those who are more awake and alert and respond favourably to the medical, nursing and rehabilitation care provided to them (Schaller et al. 2016). As their cooperation improves and their condition stabilises, patients are more likely to be able to participate in seated and out-of-bed mobilisation activities during their ICU stay (Schaller et al. 2016). Participants in the surgical group underwent surgery for cancerous diseases, complications during childbirth, renal and bowel disease and as source control for infectious disease. Their CPAx scores on admission to ICU were high which may indicate adequate physical function on admission. Change in CPAx scores for this group was relatively small during their ICU stay. The small change in CPAx scores (3.2 points) observed during the ICU stay for the surgical group may be explained by the fact that some of them had chronic disease prior to surgery which could impact on their general health status (Farhan et al. 2016). The change in CPAx scores between admission and discharge from ICU was greater (7.5 points) for the trauma group than the surgical group. Admission diagnoses in the trauma group included blunt and penetrating trauma, mostly to the abdomen, heart, lungs or neck. People who suffer traumatic injury are usually independent in their communities prior to admission, and even though some may suffer from comorbidities, these are not the primary reason for admission to hospital (Clark et al. 2013). This may explain the greater change in within-group recovery of physical function observed in the trauma group.

Corner et al. (2015) reported that a change in CPAx score of six or more can be considered a clinically meaningful change in physical function in a burns ICU population. One could therefore extrapolate that the current within-group difference in CPAx scores observed for the trauma group suggests a clinically relevant finding as both this group and those reported on by Corner et al. (2015) can be considered to be independent in functional ability prior to sustaining traumatic injury. The median CPAx score was significantly higher at ICU discharge for the surgical group than for the trauma group. A possible explanation for this finding is the small number of trauma participants admitted with fractures to the extremities in whom the extent of bone healing and permitted weight bearing would influence their level of functional participation at the time of ICU discharge.

Duration of stay in ICU and in hospital was not significantly influenced by the addition of the CPAx tool to standard physiotherapy patient management when compared to results for the historical control group. The patients however did show clinical improvements in physical function. Informal feedback from the two research assistants indicated that they found the CPAx tool enhanced their accuracy of patient assessment. They also reported that the CPAx assisted with patient care and planning, assisted with evaluation of patient progression, served as motivation for patients to participate in treatment, enhanced communication with patients and motivated them regarding patient response to treatment.

Higher CPAx scores on ICU admission, but not on ICU discharge, seemed to be associated with shorter hospital LOS. Although some studies have shown that early mobilisation in ICU is associated with shorter duration of ICU and hospital stay (Kayambu et al. 2013; Schaller et al. 2016; Stiller 2013), other studies have found no such associations (Clark et al. 2013; Morris et al. 2016) which is similar to our findings. Complications that develop postoperatively or post-injury such as postoperative pulmonary complications, wound infection or sepsis, renal insufficiency and multi-organ dysfunction may arise in some patients and influence their LOS in the unit. Such complications are not amenable to early rehabilitation interventions in ICU alone (Denehy et al. 2018). A major limitation of our study is that information on the frequency of development of postoperative or post-injury complications for participants was not collected.

Another factor that could have influenced LOS in our study is the relatively short duration of stay of participants in ICU. Participants were younger and less severely ill on admission to ICU compared to demographics reported for other ICU populations and this might account for their shorter LOS (Clark et al. 2013; Corner et al. 2015; Schaller et al. 2016). Also the large catchment area for CHBAH and hence the constant demand for ICU beds might have played a role. Clinical experience at CHBAH confirms that this high demand for beds often results in patients being discharged to the wards prematurely. Mortality rates reported for surgical patients admitted to South African ICUs can be as high as 33% for unplanned admissions into the ICU (Skinner et al. 2017). This is similar to the mortality rate identified in CHBAH ICUs during our sample size estimation, but during the trial only one person died in ICU (3.8%). The low mortality rate during the trial suggests that participants developed few life-threatening complications (confirmed by the low SOFA scores) during their ICU stay which may have resulted in quicker discharge from the unit.

Results showed that an increase in physical function level had a strong positive relationship with severity of morbidity (SOFA score) at ICU discharge. This finding was unexpected as one would assume that lower morbidity is associated with better physical functioning. The reduction in the number of participants in the trial at the time of ICU discharge because of high patient turnover through the units might have influenced the results. Alternatively one may suggest that changes in patient physiology do not immediately impact on their level of physical activity in the short term. This assumption should be tested in future trials. It is however well known that persistent inflammation and infection because of complications of critical illness and the resultant muscle weakness that ensues, impact significantly on surgical and trauma patients’ cardiovascular endurance, physical functioning and health-related quality of life long after discharge from hospital (Dinglas et al. 2017; Fan et al. 2014; Van Aswegen et al. 2011).

Limitations of this study include the following: (1) information on comorbidities (such as human immunodeficiency virus or diabetes mellitus) that participants in the intervention group experienced prior to admission to ICU was not collected; (2) change in muscle power (apart from hand-grip strength) during ICU stay was not objectively assessed in this trial. It is therefore not possible to comment on the extent of muscle weakness that participants experienced on admission to ICU or how this might have influenced levels of physical functioning during ICU stay; and lastly, (3) formal testing of adherence of physiotherapists to the implementation of the CPAx tool for patient assessment and their progression of individual needs-based patient treatment was not done and is recommended for future trials.

## Conclusion

Problem-oriented patient rehabilitation informed by the CPAx tool in two ICUs where early mobilisation forms part of standard physiotherapy care resulted in improvements in physical function for all participants during their ICU stay but did not influence ICU or hospital LOS when compared to historic controls. Higher levels of physical function at ICU admission were associated with shorter hospital LOS for this cohort. Surgical patients had significantly higher levels of physical function compared to trauma patients at ICU discharge.
